# Visual impairment in aging and cognitive decline: experience in a Memory Clinic

**DOI:** 10.1038/s41598-019-45055-9

**Published:** 2019-06-18

**Authors:** Marta Marquié, Miguel Castilla-Martí, Sergi Valero, Joan Martínez, Domingo Sánchez, Isabel Hernández, Maitée Rosende-Roca, Liliana Vargas, Ana Mauleón, Octavio Rodríguez-Gómez, Carla Abdelnour, Silvia Gil, Miguel A. Santos-Santos, Montserrat Alegret, Ana Espinosa, Gemma Ortega, Alba Pérez-Cordón, Ángela Sanabria, Natalia Roberto, Sonia Moreno-Grau, Itziar de Rojas, Rafael Simó, Andreea Ciudin, Cristina Hernández, Adelina Orellana, Gemma Monté-Rubio, Alba Benaque, Agustín Ruiz, Lluís Tárraga, Mercè Boada

**Affiliations:** 1 Alzheimer Research Center and Memory Clinic, Fundació ACE Institut Català de Neurociències Aplicades - Universitat Internacional de Catalunya (UIC), Barcelona, Spain; 2 Clínica Oftalmológica Dr. Castilla, Barcelona, Spain; 3 Department of Ophthalmology, Hospital del Mar and Hospital de l’Esperança - Parc de Salut Mar, Barcelona, Spain; 4 Centro de Investigación Biomédica en Red de Enfermedades Neurodegenerativas (CIBERNED), Instituto de Salud Carlos III, Madrid, Spain; 5 Centro de Investigación Biomédica en Red de Diabetes y Enfermedades Metabólicas Asociadas (CIBERDEM), Instituto de Salud Carlos III, Madrid, Spain; 6 Institut de Recerca Vall d’Hebron, Universitat Autònoma de Barcelona (VHIR-UAB), Barcelona, Spain

**Keywords:** Alzheimer's disease, Eye diseases

## Abstract

Visual impairment is common in people living with dementia and regular ophthalmological exams may improve their quality of life. We evaluated visual function in a cohort of elderly individuals and analyzed its association with their degree of cognitive impairment. Participants underwent neurological and neuropsychological exams, neuro-ophthalmological assessment (visual acuity, intraocular pressure, rates of past ophthalmological pathologies, use of ocular correction, treatments and surgeries) and optical coherence tomography (OCT) scan. We analyzed differences in ophthalmological characteristics among diagnostic groups. The final sample of 1746 study participants aged ≥ 50 comprised 229 individuals with Subjective Cognitive Decline (SCD), 695 with mild cognitive impairment (MCI) and 833 with Dementia (Alzheimer disease: n = 660; vascular dementia: n = 92, Lewy body dementia: n = 34; frontotemporal dementia: n = 19 and other: n = 28). Age, gender and education were used as covariates. Patients with Dementia, compared to those with SCD and MCI, presented worse visual acuity (p < 0.001), used less visual correction (p = 0.02 and p < 0.001, respectively) and fewer ophthalmological treatments (p = 0.004 and p < 0.001, respectively) and underwent fewer ocular surgeries (p = 0.009 and p < 0.001, respectively). OCT image quality worsened in parallel to cognitive decline (Dementia vs SCD: p = 0.008; Dementia vs MCI: p < 0.001). No group differences in past ophthalmological disorders or abnormal OCT findings were detected. Efforts should be made to ensure dementia patients undergo regular ophthalmological assessments to correct their visual function in order to improve their quality of life.

## Introduction

Alzheimer’s Disease (AD), the leading cause of dementia in the elderly, is a neurodegenerative condition that irreversibly impairs cognition^1^. AD symptoms progressively develop over time and include memory loss, impairment of judgment, disorientation, personality changes and emotional disturbances^2^. Visual abnormalities are also common in AD, leading to falls, confusion and difficulty with activities of daily living. In combination with visual hallucinations, poor vision can affect an individual’s autonomy and quality of life^3^. Large population studies have identified a higher prevalence of age-related visual problems among elderly people with dementia compared to the general population^4^. The burden of both visual impairment and cognitive decline is expected to rise in the near future due to the increase in life expectancy^5,6^.

AD-related neuropathological lesions have been described in all parts of the visual system^7–10^ and result in a variety of signs and symptoms. These lesions include deposition of β-amyloid plaques in the lens^7^, reduction in the number of retinal ganglion cells and thinning of the peripapillary retinal nerve fiber layer (RNFL)^8,11^, optic disc pallor, atrophy and cupping^12,13^, reduction of axons in the optic nerve^14,15^, accumulation of lipofucsin in the lateral geniculate nucleus^3^, loss of pyramidal cells in the visual cortex^9,16^, and presence of numerous cored senile plaques but few neurofibrillary tangles in the visual cortex^10,16^.

Common visual symptoms reported in AD comprise diminished color contrast sensitivity^17^, visual field defects^18^, delayed saccadic eye movements and slow pursuit movements^19^, complex visual deficits such as poor understanding of written words (impairing reading abilities)^20^, difficulties in object and shape recognition^20,21^ and problems finding objects^22^. Additionally, 20% of AD patients experience visual hallucinations, especially those with impaired vision and more severe cognitive decline^23^. Posterior cortical atrophy (PCA) is a neurodegenerative condition that affects the visual-processing pathways and is mostly associated with underlying AD pathology primarily in the visual association areas^24^. PCA manifests with visual symptoms such as difficulties in reading and driving, object ataxia, oculomotor apraxia, simultanagnosia, left hemineglect, topographical disorientation, agraphia, acalculia, visual agnosia and prosopagnosia, among others, and it can be severely disabling as affected patients behave like a blind person. Individuals suffering from other types of dementia different from AD may also exhibit significant visual problems. Dementia with Lewy bodies (DLB) is the second most frequent type of neurodegenerative dementia and is characterized by parkinsonism, cognitive fluctuations and vivid visual hallucinations co-occurring with the onset of cognitive decline^25^. Impairments in multiple visual domains have been described in DLB, such as visual construction, object and space-motion perception, line orientation perception and saccadic eye movements. In post-stroke vascular dementia, up to 30% of patients experience visual impairment^26^. Homonymous hemianopia (especially in occipital strokes) is the most reported symptom, but neglect, diplopia, ptosis, anisocoria and nystagmus have also been described. Charles-Bonnet syndrome is a common cause of visual hallucinations secondary to poor vision in elderly population^27^.

As dementia evolves, patients become less capable of describing and complaining about their visual problems^28^. Thus, it is essential to perform periodical ophthalmological screenings to assess their visual acuity, rule out common age-related eye pathologies such as open-angle glaucoma, age-related macular degeneration (AMD) and dry eye syndrome and offer appropriate vision correction or treatment if available (e.g. earlier cataract extraction), which can potentially lead to improvement of patients’ functional state.

We aimed to assess visual function and ophthalmological care received in a cohort of older adults who were attending a Memory Clinic due to cognitive decline and to determine whether their degree of cognitive impairment influenced the presence, prior detection and correction/treatment of common ocular pathologies. Evaluation was carried out by performing a neuro-ophthalmological exam and an Optical Coherence Tomography (OCT) scan.

## Methods

### Study subjects

Fundació ACE is a non-profit organization located in Barcelona, Spain that was declared a welfare institution by the Generalitat of Catalonia in 1995. The present study derives from the *Neuro-Ophthalmology Research At Fundació ACE* (NORFACE) cohort, which was founded in 2014 to search for retinal biomarkers of AD and examine the relationship between retinal changes and different types of neurodegenerative disorders^29^. Consecutive patients evaluated due to cognitive decline at Fundació ACE between January 2017 and March 2018 were enrolled in the present study. Participants were recruited from three sources: 1) the Memory Clinic, an outpatient diagnostic unit for patients with cognitive decline that has an agreement with the Catalan Public Health System, 2) *Fundació ACE*’*s Open House Initiative*^30^, a corporate social responsibility program that assesses for free the cognitive status of individuals from the community without the need of a physician’s referral, and 3) *Fundació ACE Healthy Brain Initiative* (FACEHBI)^31^, a research study with the goal of identifying biomarkers of preclinical AD in individuals with Subjective Cognitive Decline (SCD). Inclusion criteria were: age ≥ 50-yo, presence of a consensus-based clinical diagnosis about the participants’ cognitive status and ability to complete the full ophthalmological exam and OCT scan (excluding those patients with severe dementia stages, equivalent to a Global Deteriorating Scale [GDS]^32^ score > 6).

### Clinical diagnostic groups

Study participants completed neurological, neuropsychological and social evaluations at Fundació ACE. For each individual, a consensus-based diagnosis about his/her cognitive status (Dementia vs mild cognitive impairment [MCI] vs subjective cognitive decline [SCD]) was reached at the time of the study recruitment by a multidisciplinary team of professionals that included neurologists, neuropsychologists and social workers^33^. Cognitive assessment consisted of the Spanish version of the Mini-Mental State Examination (MMSE)^34,35^, the memory part of the Spanish version of the 7 Minutes test^36^, the Hachinski Ischemic Scale^37^, the Spanish version of the Neuropsychiatric Inventory Questionnaire (NPI-Q)^38^, the GDS^32^, the Clinical Dementia Rating Score (CDR)^39^, the Blessed Dementia Scale^40^ and a comprehensive neuropsychological battery of Fundació ACE (NBACE)^41,42^. Demographical information collected included age, gender and years of education.

Dementia was defined according to the DSM-V criteria^43^. MCI was defined using Petersen’s criteria^44^. SCD refers to the perception of memory or other cognitive problems without impairment on standardized cognitive tests^45^. Specifically, individuals with SCD had a CDR^39^ of 0, a preserved performance (score ≥ 27) on the MMSE^34,35^, a score of ≥8 on the Spanish Modified Questionnaire of Memory Failures in Everyday (MFE-30)^46^, and a strictly normal performance in the NBACE^41,42^.

### Neuro-ophthalmological evaluation

Study participants underwent a complete neuro-ophthalmological evaluation in parallel to the cognitive evaluation (usually the same day of the initial neurological assessment). The whole visit was performed by an optometrist and was completed in 20 mins. In order to undergo the exam, subjects had to be able to cooperate, obey simple instructions and sit still for a few minutes. The evaluation comprised: 1) review of past ophthalmological diseases, treatments and surgeries, 2) monocular visual acuity assessment with the participants wearing their habitual correction for refractive error using a pinhole occluder and the Early Treatment of Diabetic Retinopathy Study (ETDRS) chart^47,48^, 3) intraocular pressure (IOP) measurement by Icare tonometry^49^, and 4) swept source (SS) OCT scan. The visual acuity assessment was done the same way for all participants, regardless of their cognitive status and level of cooperation. Reduced visual acuity was defined as a standard LogMAR fraction scale ≤ 20/50 at 20 ft (equivalent to a fraction scale of 6/15 at 6 m and a decimal scale of 0.4) according to the Snellen scale^50^, which is in line with the North American guidelines of visual loss^51^. High IOP was defined as ≥24 mmHg using Icare Tonometry^49^. All assessments were carried out by a single optometrist and reviewed by a single ophthalmologist. Before beginning the study, the ophthalmologist trained the optometrist in the evaluation of OCT images, with the goal to differentiate normal images from abnormal findings. The ophthalmologist reviewed the ophthalmological history, ocular exam findings and OCT images from those cases in which OCT images were considered abnormal by the optometrist and came up with a diagnosis if appropriate. Of note, the diagnoses of dry and wet AMD were based on the Age-Macular Degeneration Preferred Practice Patterns guidelines from the American Academy of Ophthalmology (2015)^52^, which uses the classification of the Age-Related Eye Disease Study^53^ and a more recent classification to define the early and intermediate stages of AMD^54^. The glaucoma category was established based on the image of the head of the optic nerve (ONH), ONH OCT findings and Icare IOP measurements. If a participant presented with what is commonly called a “suspicious papilla”, an IOP > 24 mmHg, and signs of thinning of the RNFL in the OCT, the case was assigned a label of “glaucoma” based on OCT-findings. No anterior chamber OCT imaging was available. The ophthalmologist and neurologists were blind to each other’s diagnosis.

### Optical coherence tomography

Participants were imaged with a DRI OCT Triton - Swept Source (SS) OCT, software v.1.22.1 (Topcon Co. Tokyo, Japan). The OCT exam was completed in about 5–10 minutes. Both eyes were scanned separately. No pupil dilation was required. The device used to capture OCT integrates a high-quality retinograph, which acquires a color image of the fundus at the same time that OCT structural and vascular measures are obtained. These images were used as support when analyzing the cases. Retinal layer segmentation from the macular and peripapillary regions was performed using the Topcon Advanced Boundary Segmentation TM (TABS) algorithm as part of the Fast Map software^55^ (data not shown).

### Ethical considerations

This study and its informed consent were approved by the ethics committees of the *Hospital Clínic i Provincial de Barcelona* and the *Hospital Universitari Vall d’Hebron* in Barcelona, Spain, in accordance with Spanish biomedical laws (Law 14/2007, July 3^rd^, about biomedical research; Royal Decree 1716/2011, November 18^th^) and followed the recommendations of the Declaration of Helsinki. All participants signed the study informed consent (for those with moderate dementia stages, the informed consent was signed by their legal representative or family member).

### Statistical analysis

Data processing and analysis were conducted using SPSS statistical software v. 20. Alpha level was set at 0.05. Frequency analysis and measures of central tendency and dispersion were used to describe the study variables. Bootstrap was used to resample the cohort in order to obtain the 95% confidence interval (CI) for each variable. Demographic data differences among diagnostic groups (SCD vs MCI vs Dementia) were assessed with an ANOVA test for quantitative variables (age, years of education and MMSE scores) and a Chi-Square test for qualitative variables (gender). A Multinomial Logistic Regression (MLR) model including age, gender and years of education as co-variates was used to analyze the differences in ophthalmological pathologies (history of glaucoma and AMD, use of corrective lenses, past ocular surgeries and current ophthalmological treatment), visual exam (low visual acuity and high IOP) and OCT scan findings (suboptimal OCT image quality, abnormal OCT findings and newly detected ophthalmologic pathology) among the three diagnostic groups. We performed a similar MLR model within the Dementia cohort (n = 684) to analyze differences in visual acuity across GDS sub-groups, including age, gender and years of education as co-variates. Additionally, we performed a Linear Regression Analysis adjusted by age, gender and years of education within the Dementia cohort to explore the relationship between MMSE scores and visual acuity.

## Results

### Demographical characteristics of the cohort

1813 individuals were assessed between January 2017 and March 2018, had a consensus-based clinical diagnosis and underwent ophthalmological exam and OCT scan. Of those, we excluded 22 individuals due to age < 50 and 45 due to incomplete ophthalmological exam and/or OCT scan. Detailed reasons for the exclusions are reported in Supplementary Table S1.

The final study cohort comprised 1746 individuals. Participants were sub-classified into 3 clinical groups according to their cognitive stage: SCD, MCI and Dementia. Due to the low number of cases observed in a few variables and in order to be able to run the appropriate statistical analysis, we simplified the dataset and dichotomized the subcategories within each ophthalmological variable (present/absent or normal/abnormal). A description of the study sample used for statistical analysis is shown in Tables 1–3, while detailed information is shown in Supplementary Tables 2–4.

**Table 1:** Demographic characteristics of the study cohort.

n	Whole cohort	SCD	MCI	Dementia	p
	1746	229	684	866	
Age, mean (SD)	76.7 ± 9.2	66.7 ± 7.7	74.2 ± 8.01	81.4 ± 7.2	<0.001*
Females, n (%)	1149 (65.8%)	149 (65.1%)	422 (61.7%)	579 (69.4%)	0.01*
Years of education, mean (SD)	7.2 (4.4)	11.69 (3.7)	7.27 (4.1)	5.9 (3.9)	<0.001*
MMSE, mean (range)	22.9 (0–30)	29.2 (25–30)	26.0 (16–30)	18.7 (0–30)	<0.001*

Statistical significance was set up at p < 0.05. A Chi-Square test was used to compare gender differences among groups. 1-factor ANOVA was used to compare age, years of education and MMSE scores differences among groups.

MCI = mild cognitive impairment; MMSE = Mini-Mental State Examination; OCT = optical coherence tomography; SCD = subjective cognitive decline; SD = standard deviation.

**Table 2:** Past ophthalmological history of the study cohort.

	Whole cohort (n = 1746)	SCD (n = 229)	MCI (n = 684)	Dementia (n = 833)
Glaucoma, n (%, [95% CI])	137 (7.8% [6.5–9.1])	13 (5.7% [3.1–8.7])	60 (8.8% [6.6–10.8])	64 (7.7% [5.9–9.7])
AMD, n (%, [95% CI])	71 (4.1% [3.2–5])	4 (1.7% [0.4–3.5])	27 (3.9% [2.5–5.4])	40 (4.8% [3.5–6.2])
Corrective lenses, n (%, [95% CI])	1583 (90.7% [89.3–92])	218 (95.2% [92.1–97.8])	643 (94% [92.1–97.8])	722 (86.7% [84.2–89.1])
Past ocular surgeries, n (%, [95% CI])	728 (41.7% [39.3–44.2])	59 (25.8% [20.1–31.4])	276 (40.4% [36.5–44])	393 (47.2% [44.1–50.4])
Current ophthalmological treatment, n (%, [95% CI])	538 (30.8% [28.5–31.8])	72 (31.4% [25.3–37.6])	235 (34.4% [31–38])	231 (27.7% [24.7–30.7])

AMD = age-related macular degeneration; CI = confidence interval; MCI = mild cognitive impairment; SCD = subjective cognitive decline.

**Table 3:** Ophthalmological exam and OCT findings of the study cohort.

	Whole cohort (n = 1746)	SCD (n = 229)	MCI (n = 684)	Dementia (n = 833)
Low visual acuity, n (%, [95% CI])	453 (25.9% [24–27.9])	14 (6.1% [3.5–9.2])	131 (19.2% [15.9–22.1])	308 (37% [33.9–40.3])
High IOP, n (%, [95% CI])	110 (6.3% [5.2–7.4])	12 (5.2% [2.6–7.9])	44 (6.4% [4.7–8.3])	54 (6.5% [4.8–8.3])
Suboptimal OCT image quality, n (%, [95% CI])	173 (9.9% [8.6–11.3])	1 (0.4% [0–1.3])	42 (6.1% [4.2–7.9])	130 (15.6% [13.1–18.1])
Abnormal OCT findings, n (%, [95% CI])	315 (18% [16.2–19.8])	17 (7.4% [4.4–10.9])	123 (18% [15.2–20.8])	175 (21% [18.2–23.8])
Newly detected ophthalmologic pathology, n (%, [95% CI])	213 (12.2% [10.7–13.7])	15 (6.6% [3.5–10.4])	73 (10.7% [8.5–13])	125 (15% [12.5–17.5])

CI = confidence interval; IOP = intraocular pressure; MCI = mild cognitive impairment; OCT = optical coherence tomography; SCD = subjective cognitive decline.

Demographic information of the cohort is displayed in Table 1. Mean age was 76.7 ± 9.17 years and 1149 participants (65.8%) were female. 229 individuals (13.1%) were classified as SCD, 684 (38.2%) as MCI and 833 (47.7%) as Dementia. Within the Dementia group, 660 patients were diagnosed as AD^56^, 92 as vascular dementia^57^, 34 as Lewy body spectrum dementia^25^ 19 as frontotemporal dementia (FTD)^58^, and 28 as other dementia types (data not shown). Within the Dementia group we employed the GDS^32^ to sub-classify patients into three categories: Mild (GDS 4, n = 498, 59.78%), Moderate (GDS 5, n = 294, 29.89%) and Moderate-Severe (GDS 6, n = 41, 4.9%).

We detected significant differences among diagnostic groups in terms of age (SCD 66.7 ± 7.7, MCI 74.2 ± 8.0, Dementia 81.4 ± 7.2; ANOVA p < 0.001), gender (females: SCD 149 (65.1%), MCI 422 (61.7%), Dementia 579 (69.4%); ANOVA p = 0.01) and years of education (SCD 11.7 ± 3.7, MCI 7.3 ± 4.1, Dementia 5.9 ± 3.9; ANOVA p < 0.001, Table 1). Consequently age, gender and years of education were added as co-variates in the analyses. As expected, MMSE^34,35^ scores were significantly different among the diagnostic groups (SCD 29.2, MCI 26.0, Dementia 18.3; ANOVA p < 0.001; Table 1).

### Past ophthalmological history

Data from the participants’ past ophthalmological history are detailed in Tables 2 and 4 and Supplementary Table S2.

**Table 4:** Diagnostic group differences in past ophthalmological pathologies and treatments.

	OR (95% CI)	p
Glaucoma		
Dementia vs SCD	0.90 (0.43–1.92)	0.79
Dementia vs MCI	0.72 (0.48–1.07)	0.45
SCD vs MCI	0.79 (0.39–1.60)	0.52
AMD		
Dementia vs SCD	0.97 (0.29–3.25)	0.96
Dementia vs MCI	0.71 (0.41–1.23)	0.22
SCD vs MCI	0.73 (0.23–2.38)	0.61
Corrective lenses		
Dementia vs SCD	0.37 (0.17–0.84)	0.02*
Dementia vs MCI	0.46 (0.30–0.70)	<0.001*
SCD vs MCI	1.23 (0.56–2.70)	0.61
Past ocular surgeries		
Dementia vs SCD	0.56 (0.37–0.87)	0.01*
Dementia vs MCI	0.60 (0.47–0.77)	<0.001*
SCD vs MCI	1.06 (0.71–1.59)	0.76
Current ophthalmological treatment		
Dementia vs SCD	0.55 (0.37–0.82)	0.01*
Dementia vs MCI	0.59 (0.46–0.76)	<0.001*
SCD vs MCI	1.08 (0.75–1.57)	0.68

A Multinomial Logistic Regression model including age, gender and years of education as co-variates was used to analyse group differences in past ophthalmological pathologies and treatments. For each comparison, the first listed group (Dementia, MCI or SCD) acted as reference. Statistical significance was set-up at p < 0.05.

AMD = age-related macular degeneration; CI = confidence interval; MCI = mild cognitive impairment; OR = odds ratio; SCD = subjective cognitive decline.

137 individuals (7.8%) had a previous diagnosis of open-angle glaucoma (13 (5.7%) with SCD, 60 (8.8%) with MCI and 64 (7.7%) with Dementia), while 71 participants (4.1%) had a previous diagnosis of age-related macular degeneration (AMD) (4 (1.7%) with SCD, 27 (3.9%) with MCI and 40 (4.8%) with Dementia). After adjusting for age, gender and years of education, no statistical differences in terms of open-angle glaucoma or AMD previous diagnosis were identified among diagnostic groups.

Most of the study participants (n = 1583, 90.7%) were currently using corrective lenses (218 (95.2%) with SCD, 643 (94%) with MCI and 722 (86.7%) with Dementia). After adjusting for age, gender and years of education, data showed that patients with Dementia were less likely to be currently using lenses compared to individuals with SCD (OR = 0.37, 95% CI = 0.17–0.84, p = 0.02) and MCI (OR = 0.46, 95% CI = 0.30–0.70, p < 0.001), while there were no significant differences between MCI and SCD participants.

728 participants (41.7%) had undergone ocular surgery (650 [89.29%] for cataract extraction, 18 [2.47%] due to glaucoma, 11 [1.51%] due to retinal detachment and 49 [6.73%] for other reasons: 59 (25.8%) with SCD, 276 (40.4%) with MCI and 393 (47.2%) with Dementia). After adjusting for age, gender and years of education, patients in the Dementia group showed significant lower odds of having undergone ocular surgery compared to those with SCD (OR = 0.56, 95% CI = 0.37–0.87, p = 0.01) and MCI (OR = 0.60, 95% CI = 0.47–0.77, p < 0.001), while there were no significant differences between MCI and SCD groups.

538 participants (30.8%) were taking topical ophthalmological treatment (72 (31.4%) with SCD, 235 (34.4%) with MCI and 393 (27.7%) with Dementia). After adjusting for age, gender and years of education, the analysis showed that patients with Dementia were less likely to receive ophthalmological treatment than those in the SCD group (OR = 0.55, 95% CI = 0.37–0.82, p = 0.004) and the MCI group (OR = 0.59, 95% CI = 0.46–0.76, p < 0.001).

### Ophthalmological exam findings

Data from the participants’ ophthalmological exam are detailed in Tables 3 and 5 and Supplementary Table S3.

**Table 5:** Diagnostic group differences in ophthalmological exam and OCT findings.

	OR (95% CI)	p
Reduced visual acuity:		
Dementia vs SCD	3.36 (1.77–6.39)	<0.001*
Dementia vs MCI	1.61 (1.24–2.10)	<0.001*
SCD vs MCI	0.48 (0.25–0.90)	0.02*
High Intraocular pressure:		
Dementia vs SCD	0.85 (0.40–1.83)	0.68
Dementia vs MCI	1.08 (0.69–1.70)	0.73
SCD vs MCI	1.27 (0.61–2.62)	0.52
Suboptimal OCT image quality:		
Dementia vs SCD	14.95 (2.01–111.03)	0.01*
Dementia vs MCI	2.01 (1.36–2.98)	<0.001*
SCD vs MCI	0.14 (0.02–1.00)	0.05
Abnormal OCT findings:		
Dementia vs SCD	1.23 (0.67–2.25)	0.51
Dementia vs MCI	0.81 (0.61–1.07)	0.13
SCD vs MCI	0.66 (0.36–1.18)	0.16
Newly detected ophthalmological pathologies:		
Dementia vs MCI	1.23 (0.64–2.38)	0.54
Dementia vs SCD	1.07 (0.77–1.50)	0.69
SCD vs MCI	0.87 (0.46–1.66)	0.67

A Multinomial Logistic Regression model including age, gender and years of education as co-variates was used to analyse group differences in ophthalmological exam and OCT scan findings. For each comparison, the first listed group acted as the reference one. Statistical significance was set-up at p < 0.05.

CI = confidence interval; MCI = mild cognitive impairment; OCT = optical coherence tomography; OR = odds ratio; SCD = subjective cognitive decline.

453 participants (25.9%) suffered from low visual acuity as assessed by the ETDRS chart (14 (6.1%) with SCD, 131 (19.2%) with MCI and 308 (37%) with Dementia). After adjusting for age, gender and years of education, data showed that patients with Dementia were almost 3.5-times more likely to present with reduced visual acuity compared to those in the SCD group (OR = 3.36, 95% CI = 1.77–6.39, p < 0.001) and over 1.5 times compared to those in the MCI group (OR = 1.61, 95% CI = 1.77–6.39, p < 0.001). Additionally, individuals with SCD had half the odds of presenting with reduced visual acuity than MCI patients (OR = 0.48, 95% CI = 0.25–0.90, p = 0.023). We analyzed differences in visual acuity within the Dementia cohort according to patients’ GDS status (GDS = 4: mild dementia, GDS = 5: moderate dementia and GDS = 6: moderate-severe dementia)^32^ (Supplementary Table S4). After adjusting for age, gender and years of education, data showed that patients with mild Dementia had 30% lower odds of presenting with reduced visual acuity compared to those with moderate Dementia (OR = 0.68; 95% CI = 0.50–0.93, p = 0.01). Patients with moderate-severe Dementia did not show significant differences in visual acuity compared to those with mild and moderate Dementia. Additionally, we explored the relationship within MMSE^34^ scores and visual acuity within the Dementia group adjusted by age, gender and years of education. Visual acuity had a statistical marginal effect on MMSE scores (p = 0.05), such as low visual acuity was associated to lower MMSE scores (data not shown).

Icare tonometry^49^ measurements showed that 110 participants (6.3%) had elevated IOP values (12 (5.2%) with SCD, 44 (6.4%) with MCI and 54 (6.5%) with Dementia). No statistical differences in terms of IOP were detected among diagnostic groups after adjusting for age, gender and years of education.

In 173 participants (9.9%) OCT scan images had suboptimal quality (1 (0.4%) with SCD, 42 (6.1%) with MCI and 130 (15.6%) with Dementia). After adjusting for age, gender and years of education, the analysis demonstrated that patients with Dementia were at least 2-times more likely to show worse OCT image quality compared to SCD participants (OR = 14.95, 95% CI = 2.01–111.03, p < 0.008) and at least 1.4-times more likely compared to those with MCI (OR = 2.01, 95% CI = 1.36–2.98, p < 0.001). OCT image quality differences between SCD and MCI groups were borderline significant, with patients in the MCI group showing worse image quality than their peers with SCD (OR = 0.14, 95% CI = 0.02–1.00, p = 0.05).

In 315 participants (18%) OCT images were considered to be pathological (17 (7.4%) with SCD, 123 (18%) with MCI and 175 (21%) with Dementia) but no significant differences were detected among groups after adjusting for age, gender and years of education. The most frequent OCT-based diagnosis was posterior pole deformation due to posterior staphyloma/myopia magna (n = 70) followed by epiretinal membrane (n = 63), image suggestive of glaucomatous pathology (n = 60), dry AMD (n = 49), retinal pigmented epithelium abnormality (n = 47), other maculopathies (n = 30), other neuropathies (n = 19) and wet AMD (n = 19).

After completing the study’s neuro-ophthalmological exam and OCT scan, a new ophthalmological pathology was detected in 213 participants (12.2%) (15 (6.6%) with SCD, 73 (10.7%) with MCI and 125 (15%) with Dementia). No statistical differences were detected among groups after adjusting for age, gender and years of education. The types of new ophthalmological pathologies identified were as follows: posterior pole deformation due to posterior staphyloma/myopia magna (n = 56), epiretinal membrane (n = 42), retinal pigmented epithelium abnormality (n = 27), dry AMC (n = 23), other maculopathies (n = 13), image suggestive of glaucomatous pathology (n = 12) and wet AMD (n = 10). Those participants in whom a new pathology was detected were recommended to consult with an ophthalmologist for a full evaluation.

## Discussion

In the present study we analyzed visual function, ophthalmologic pathologies and care received and OCT scan findings in a cohort of 1746 individuals evaluated at a Memory Clinic. We detected that patients living with Dementia presented with worse visual acuity, were less likely to receive adequate visual health care and exhibited suboptimal OCT scan image quality compared to those with MCI and SCD.

First, we examined the prevalence of common age-related ocular pathologies in the three study groups. After adjusting for age, gender and years of education, we did not detect a higher prevalence of open-angle glaucoma or AMD in patients with Dementia compared to those with SCD and MCI. These two ocular pathologies are leading causes of visual loss in older adults of developed countries and share with AD common risk factors, a chronic decline of function and histopathologic features, although their relationship with dementia is not fully understood^59–62^. Several studies have detected a two-to-three fold increase of glaucoma prevalence in patients with AD^59,63^ but there are conflicting results about whether patients with glaucoma have a higher risk of developing AD^61,64^. Most of the studies that analyze the relationship of retinal thickness changes assessed by OCT and cognitive decline consistently exclude cases with a previous diagnosis of open-angle glaucoma or AMD, as retinal thinning has been reported in these entities and could interfere with the measurement of that associated with AD neurodegeneration itself. It is important to note that in our cohort, as opposed to other studies, the Dementia group was not only comprised of AD patients (79.23%) but it also included patients with a diagnosis of Vascular Dementia, DLB or FTD, among others. The relation of non-AD dementia types with glaucoma is relatively understudied compared to AD and there is little data about glaucoma prevalence among those disorders. Also, our control group consisted of individuals with SCD who consulted for cognitive problems at the Memory Clinic or Open House Initiative program, instead of healthy subjects from the general population. Although individuals with SCD show unimpaired performance on formal neuropsychological testing, there is increasing evidence that SCD may represent the first symptomatic manifestation of AD^45^.

Further analysis uncovered that patients with Dementia were less likely to receive ophthalmologic health care than their peers with SCD and MCI: despite being significantly older, they were less likely to use corrective lenses, received fewer ophthalmological treatments and had lower rates of ocular surgery. Of note, unadjusted data suggested that rates of surgeries increased parallel to the degree of cognitive impairment (SCD: 25.8% vs MCI: 40.4% vs Dementia: 47.2%), but after accounting for age, gender and years of education, the analysis revealed that Dementia patients had in fact undergone fewer surgeries compared to the MCI and SCD groups. Cataract extraction was the most frequent ocular surgery performed in our sample (>89%) and it is known that it can often correct refractive errors. We acknowledge that this fact could have some impact in our data about the use of corrective lenses. Overall, these data suggest that despite the high prevalence of visual impairment in elderly patients with dementia, it is diagnosed or treated less often than in people without dementia^65^. As health care is universal and free of cost in Spain, this fact mainly excludes economic reasons for the differences detected among groups. One possible explanation could be the communication difficulties that patients with dementia experience as their cognition worsens, making them less able to report or ask support for their visual deficits. Regarding this issue, in Spain there are no specific guidelines for the ophthalmological evaluation and treatment of people with dementia. Studies show that most patients with dementia residing in long-term care facilities do not regularly use their prescription glasses, or those are no longer accurate^28^. In general, as dementia progresses, caregivers tend to adopt a non-invasive attitude about patients’ healthcare, to avoid surgical procedures and to seek less medical consultations not directly related to dementia, especially in those patients with more advanced stages of the disease and baseline psychotic symptoms^66^. In specific comorbidities, such as sensory deficits, this is known to be counter-productive, as the association of visual impairment and dementia in elderly population leads to poor quality of life, anxiety^67^, isolation^68^ and increased mortality rate^69^. Lastly, the phenomenon of “diagnostic overshadowing” could be affecting the visual care received by patients with dementia^70^. This term refers to the tendency of clinicians to overlook and underdiagnose medical co-morbidities in patients with mental retardation, psychiatric illnesses and dementia.

In the ophthalmological exam performed to the study participants, we detected that almost 40% of Dementia patients presented low visual acuity despite wearing their habitual correction for refractive errors. Visual acuity worsened in parallel to cognitive decline, as patients with Dementia were 3.4 and 1.6 times more likely to present worse visual acuity than those with SCD and MCI, respectively. Interestingly, this was not associated with previously diagnosed ocular pathologies or abnormal OCT findings. In our cohort, patients with Dementia were less likely to wear corrective lenses, to take ophthalmologic treatments and to undergo ocular surgery, as discussed above, and this could be influencing their visual acuity. Besides, complex visual deficits associated to parietal and occipital cortices damage that occur in AD^20,22^, such as impairment of reading abilities, could interfere with their performance in the ETDRS chart. Several studies have detected an inverse association between cognitive decline and visual acuity^71–73^. Recently, the Salisbury Eye Evaluation Study reported that visual impairment measured by the ETDRS chart was inversely associated with cognitive function quantified by MMSE scores both cross-sectionally and longitudinally in a large population-based sample of older US adults without dementia at baseline^73^. The Prevalence of Visual Impairment in Dementia (PrOVIDe) study is a large cross-sectional research project in the United Kingdom among 700 patients with dementia aged 60–89 years that reported a prevalence of visual impairment of 16.3% (as defined by standard logMAR < 6/18) or 32.5% (logMAR < 6/12) while participants were using their habitual visual correction. 45% of cases of visual impairment were correctable with new glasses, and almost 50% of the remaining cases were associated with cataracts (and thus potentially remediable). Moreover, undercorrected/uncorrected visual impairment was higher in residents of nursing homes compared to those living in the community. 22% of participants reported not having had a sight test in the previous two years (including 19 participants that had not been tested in the last ten years). 16% of participants could not read standard newspaper-size print with their current visual correction. The study, though, found no evidence of differences in management of visual impairment in people with dementia compared to elderly population in general^4^.

OCT image quality was also inversely related to the degree of cognitive decline. These data are in agreement with our group’s recent study^29^ which quantified peripapillary RNFL thickness in 930 individuals including cognitively healthy, amnestic-type MCI^74^ and AD, and showed that OCT image quality was the most influential factor determining RNFL variability, while there were no significant differences in RNFL thickness among clinical groups. Although OCT scan is considered a non-invasive and fast test, its completion requires minimum cooperation. Patients with moderate/advanced stages of Dementia are less able to follow directions and remain still, thus affecting the image quality achieved. This result may have relevant implications for OCT studies in aging and dementia, and suggests that data correction for image quality should be implemented in OCT studies.

Finally, we assessed OCT scan pathologic findings and newly detected ophthalmologic pathologies and found that there were no significant differences among groups. Unexpectedly, posterior pole deformation due to posterior staphyloma/myopia magna was the most frequent OCT-based diagnosis detected in our cohort (n = 70). There are several factors that could explain it. First, we did not systematically record myopia magna or alterations in the eyeball shape as a diagnosis in the participant’s past ophthalmological history. Secondly, we only recorded the main OCT-based diagnosis, and given the great deformation of the posterior pole that this condition implies, it tends to cancel out other possible diagnoses, such as epiretinal membrane or AMD. Lastly, cases with major posterior pole alterations that do not fall within other more specific categories would also be included in this group. Among the 70 participants in which posterior pole deformation was detected, 37 of them had low visual acuity and 59 were using visual correction. Additionally, it is important to emphasize that a clinical diagnosis of glaucoma would require a face-to-face visit with an ophthalmologist, supported by functional type tests (e.g. visual fields evaluation), examination of the eye fundus by indirect ophthalmoscopy and/or stereoscopic pictures, and often several longitudinal visits to reach a firm diagnosis. Given the design of the study, we could only establish a suspicion of glaucoma, but not determine if it was primary open-angle glaucoma or a secondary glaucoma. We cannot rule out that if we had performed such additional tests, the number of glaucoma diagnoses would have been higher in our cohort.

The main strengths of our study are the large sample size, the consensus-based clinical diagnosis and the double-blind nature of the study. Our study had also several limitations. First of all, we did not perform a thorough ophthalmological testing covering the assessment of eye movements, reading speed or color vision, features that are known to be abnormal among patients with dementia. Diabetes status and review of signs of diabetes retinopathy were also not recorded. Furthermore, the distribution of cognitive stages within our Dementia group does not reflect the reality of the Memory Clinic or the general population, as patients with advanced stages of dementia (GDS^32^ > 6) were excluded from the study as they were not able to cooperate in the ophthalmologic exam and OCT scan. Thus, our study consists of a convenience sample instead of a pure observational/ecological one. On the other hand, this makes the study results even stronger, as even though our Dementia group included mostly mild and moderate cases, we were still able to uncover significant differences compared to MCI and SCD groups. Another limitation of the study is that we did not have access to private medical records and in some cases we may have missed relevant clinical information. Additionally, we did not perform a follow-up of those cases in which we detected abnormal OCT scan findings and were recommended to consult with an ophthalmologist.

To conclude, our study showed that in a large cohort of elderly individuals evaluated due to cognitive decline in a Memory Clinic, those diagnosed with Dementia, compared to their peers with SCD and MCI, presented with worse visual function, were less likely to receive visual healthcare and had suboptimal OCT image quality, independently of their age, gender and years of education. Both visual impairment and cognitive decline are major health issues in older adults that have significant adverse effects in their quality of life and threaten the sustainability of public health care systems. Thus, efforts should be made to guarantee regular ophthalmological assessments and optimize visual function in elderly patients living with dementia in order to improve their quality of life, avoid isolation and reduce their caregivers’ burden.

## Supplementary information

Supplementary information

## Data Availability

The datasets generated and/or analyzed during the current study are available from the corresponding author on reasonable request.
