# Supplementary material for: Visual impairment in aging and cognitive decline: experience in a Memory Clinic
Source: Sci Rep. 2019 Jun 18;9:8698. doi: 10.1038/s41598-019-45055-9 (PMC6581941; doi:10.1038/s41598-019-45055-9)
Supplement: Supplementary file 1 — Supplementary information [file 41598_2019_45055_MOESM1_ESM.pdf]

Visual impairment in aging and cognitive decline: experience in a Memory Clinic

Marta Marquié, MD, PhD; Miguel Castilla-Martí, MD, PhD; Sergi Valero, PhD; Joan Martínez, BS; Domingo Sánchez, MD; Isabel Hernández, MD, PhD; Maitée Rosende-Roca, MD; Liliana Vargas, MD; Ana Mauleón, MD; Octavio Rodríguez-Gómez, MD; Carla Abdelnour, MD; Silvia Gil, MD, PhD; Miguel A. Santos-Santos, MD, PhD; Montserrat Alegret, PhD; Ana Espinosa, PhD; Gemma Ortega, PhD; Alba Pérez-Cordón, MSc; Ángela Sanabria, PhD; Natalia Roberto, MSc; Sonia Moreno-Grau, MSc; Itziar De Rojas, MSc; Rafael Simó, MD, PhD; Andreea Ciudin, MD, PhD; Cristina Hernández, PhD; Adelina Orellana, PhD; Gemma Monté-Rubio, PhD; Alba Benaque, MD, MPH; Agustín Ruiz, MD, PhD; Lluís Tárraga, MSc; and Mercè Boada, MD, PhD

Supplementary Table 1. Reasons for exclusion from the study

**Reasons**	**n**
Age<50	22
Incomplete neuro-ophthalmological exam and /or OCT scan:	45
1. Absence of visual acuity assessment	26
- *Lack of cooperation*	26
2. Absence of IOP assessment	15
- *Lack of cooperation*	5
- *Use of contact lenses*	6
- *Others*	4
3. Incomplete OCT scan:	11
- *Lack of cooperation*	3
- *Wheelchair use*	1
- *Others*	7

Note: These reasons are not mutually exclusive.
IOP = intraocular pressure; OCT = optical coherence tomography.

Supplementary Table 2. Detailed past ophthalmological history

	Whole cohort (n=1746)	SCD (n=229)	MCI (n=684)	Dementia (n=833)
Glaucoma, No. (%, [95% CI])	137 (7.8% [6.5-9.1])	13 (5.7% [3.1-8.7])	60 (8.8% [6.6-10.8])	64 (7.7% [5.9-9.7])
AMD, No. (%, [95% CI])	71(4.1% [3.2-5])	4 (1.7% [0.4-3.5])	27 (3.9% [2.5-5.4])	40(4.8% [3.5-6.2])
Corrective lenses, No. (%, [95% CI]):				
No	163 (9.3% [8-10.7])	11 (4.8% [2.2-7.9])	41 (6% [4.2-7.7])	111 (13.3% [10.9-15.8])
Distance viewing	103 (5.9% [4.9-7])	15 (6.6% [3.5-10])	35 (5.1% [3.5-6.7])	53 (6.4% [4.7-8])
Reading	556 (31.8% [29.4-34.1])	60 (26.2% [20.1-32.3])	226 (33% [29.5-36.7])	270 (32.4% [29.3-35.7])
Bifocal	127 (7.3% [6.1-8.4])	0	34 (5% [3.2-6.6])	93 (11.2% [9.1-39.9])
Progressive	797 (45.6% [43.3-48.2])	143 (62.4% [56.3-69])	684 (50.9% [46.8-54.5])	306 (36.7% [33.5-39.9])
Past ocular surgeries, No. (%, [95% CI]):				
No	1018 (58.3% [55.9-60.6])	170 (74.2% [68.1-79.9])	408 (59.6% [56.1-63.1])	440 (52.8% [49.2-56.3])
Cataract	650 (37.2% [34.9-39.5])	41 (17.9% [12.8-23.2])	237 (34.5% [31.3-38.1])	372 (44.7% [41.3-48.1])
Glaucoma	18 (1% [0.2-0.6%])	3 (1.3% [0-3.1])	10 (1.5% [0.6-2.3])	5 (0.6% [0.1-1.2])
Retinal detachment	11 (0.6% [0.3-1])	3 (1.3% [0-3.1])	5 (0.7% [0.1-1.5])	3 (0.4% [0-0.8])
Others	49 (2.8% [2.1-3.6])	12 (5.2% [2.6-8.4])	24 (3.5% [2.2-5.1])	13 (1.6% [0.8-2.5])
Current ophthalmological treatment, No. (%, [95% CI]):				
No	1208 (69.2% [67-71.3])	157 (68.6% [62.8-74.7])	449 (65.6% [62-69.2])	602 (72.3% [69.3-75.4])
Lubricant	347 (19.9% [17.9-21.8])	55 (24% [18.6-29.5])	157 (23% [19.6-26.2])	135 (16.2% [13.5-18.7])
Hypotensive	129 (7.4% [6.2-8.8])	12 (5.2% [2.6-8])	54 (7.9% [5.9-10])	63 (7.6% [5.8-9.5])
Others	62 (3.6%	5 (2.2%	24 (3.5%	33 (4%

	[2.7-4.5]	[0.4-4.3])	[2.2-5])	[2.7-5.4])

AMD = age-related macular degeneration; CI = confidence interval; MCI = mild cognitive impairment; SCD = subjective cognitive decline.

Supplementary Table 3. Detailed ophthalmological exam and OCT findings

	**Whole cohort (n=1746)**	**SCD (n=229)**	**MCI (n=684)**	**Dementia (n=833)**
Visual acuity, No. (%, [95% CI]):				
Normal	1293(74.1% [72-76])	215(93.9% [90.4-96.9])	553 (80.8% [77.8-83.8])	553 (63% [59.7-66.1])
Low unilaterally	299 (17.1% [15.4-18.9])	13 (5.7% [2.6-9.2])	86 (12.6% [10.1-15.1])	200 (24% [21.1-27])
Low bilaterally	154 (8.8% [7.4-10.3])	1 (0.4% [0-1.3])	45 (6.6% [4.8-8.6])	108 (13% [10.7-15.1])
Intraocular pressure, No. (%, [95% CI]):				
Normal	1636 (93.7% [92.6-94.8])	217 (94.8% [91.7-97.4])	640 (93.6% [91.5-95.3])	779 (93.5% [91.8-95.1])
High unilaterally	81 (4.6% [3.7-5.6])	11 (4.8% [2.2-7.4])	31 (4.5% [3.1-6.3])	39 (4.7% [3.2-6.1])
High bilaterally	29 (1.7% [1.1-2.3])	1 (0.4% [0-1.3])	13 (1.9% [1-2.9])	15 (1.8% [1-2.8])
OCT image quality, No. (%, [95% CI])::				
Good	1573 (90.1% [88.6-91.5])	228 (99.6% [98.7-100])	642 (93.9% ([91.8-95.6])	703 (84.4% [82-86.9])
Regular	91 (5.2% [88.6-91.5])	0 (0%)	27 (3.9% [2.5-5.6])	64 (7.7% [6-9.7])
Bad	82 (4.7% [3.7-5.7])	1 (0.4% [0-1.3%])	15 (2.2% [1.2-3.4])	66 (7.9% [6-9.7])
OCT findings, No. (%, [95% CI]):				
Epiretinal membrane	63 (3.6% [2.7-4.5])	6 (2.6% [0.9-4.8])	25 (3.7% [2.3-5.1])	32 (3.8% [2.6-5.1])
Retinal pigmented epithelium abnormality	47 (2.7% [2-3.5])	0	17 (2.5% [1.5-3.7])	30 (3.6% [2.4-4.9])
Dry AMD	49 (2.8% [2.1-3.6])	1 (0.4% [0-1.3])	14 (2% [1-3.2])	34 (4.1% [2.9-5.5]
Other maculopathies	30 (1.7% [1.1-2.3])	1 (0.4% [0-1.3])	7 (1% [0.4-1.8])	22 (2.6% [1.6-3.7])
Wet AMD	19 (1.1% [0.6-1.6])	1 (0.4% [0-1.3])	6 (0.9% [0.3-1.8])	12 (1.4% [0.7-2.3])
Glaucoma	60 (3.4% [2.6-4.3])	2 (0.9% [0-2.2])	34 (5% [3.4-6.6])	24 (2.9% [1.8-4.1])
Other neuropathies	19 (1.1%	0	9 (1.3%	10 (1.2%

	[0.6-1.6])		[0.6-2.2])	[0.5-2])
Posterior staphyloma/myopia magna	71 (4.1% [3.2-5])	7 (3.1% [0.9-5.3])	23 (3.4% [2-4.8])	41 (4.9% [3.6-6.5])
Newly detected ophthalmologic pathology, No. (%, [95% CI])	213 (12.2% [10.7-13.7])	15 (6.6% [3.5-10.4])	73 (10.7% [8.5-13])	125 (15% [12.5-17.5])

AMD = age-related macular degeneration; CI = confidence interval; IOP = intraocular pressure; MCI = mild cognitive impairment; OCT = optical coherence tomography; SCD = subjective cognitive decline.

Supplementary Table 4. GDS group differences in visual acuity within the Dementia cohort

	**OR (95% CI)**	**p**
Reduced visual acuity:		
GDS 4 vs GDS 5	0.69 (0.50-0.93)	0.01*
GDS 4 vs GDS 6	0.75 (0.39-1.44)	0.38
GDS 6 vs GDS 5	0.92 (0.47-1.79)	0.80

A Multinomial Logistic Regression model including age, gender and years of education as co-variates was used to analyze GDS group differences in visual acuity within the Dementia cohort. For each comparison, the first listed group (GDS 4, 5 or 6) acted as reference. Statistical significance was se-up at $p<0.05$.

Note: GDS=4: mild dementia; GDS=5: moderate dementia; GDS=6: moderate-severe dementia.

CI = confidence interval; GDS = Global Deterioration Scale; OR = odds ratio.
